# Unexpected High Diversity of Galling Insects in the Amazonian Upper Canopy: The Savanna Out There

**DOI:** 10.1371/journal.pone.0114986

**Published:** 2014-12-31

**Authors:** Genimar R. Julião, Eduardo M. Venticinque, G. Wilson Fernandes, Peter W. Price

**Affiliations:** 1 Coordenação de Ecologia, Instituto Nacional de Pesquisa da Amazonia (INPA), Manaus, Amazonas, Brazil; 2 Fiocruz Rondônia, Laboratório de Entomologia, Porto Velho, Rondônia, Brazil; 3 Departamento de Ecologia, CB/Universidade Federal do Rio Grande do Norte, Campus Universitário, Lagoa Nova, Natal, RN, Brazil; 4 Ecologia Evolutiva e Biodiversidade/DBG, C P 486, ICB/Universidade Federal de Minas Gerais (UFMG), Belo Horizonte, Minas Gerais, Brazil; 5 Department of Biological Sciences, Northern Arizona University, Flagstaff, Arizona, United States of America; University of Tours, France

## Abstract

A relatively large number of studies reassert the strong relationship between galling insect diversity and extreme hydric and thermal status in some habitats, and an overall pattern of a greater number of galling species in the understory of scleromorphic vegetation. We compared galling insect diversity in the forest canopy and its relationship with tree richness among upland *terra firme*, *várzea,* and *igapó* floodplains in Amazonia, Brazil. The soils of these forest types have highly different hydric and nutritional status. Overall, we examined the upper layer of 1,091 tree crowns. Galling species richness and abundance were higher in *terra firme* forests compared to *várzea* and *igapó* forests. GLM-ANCOVA models revealed that the number of tree species sampled in each forest type was determinant in the gall-forming insect diversity. The ratio between galling insect richness and number of tree species sampled (GIR/TSS ratio) was higher in the *terra firme* forest and in seasonally flooded *igapó*, while the *várzea* presented the lowest GIR/TSS ratio. In this study, we recorded unprecedented values of galling species diversity and abundance *per* sampling point. The GIR/TSS ratio from *várzea* was approximately 2.5 times higher than the highest value of this ratio ever reported in the literature. Based on this fact, we ascertained that *várzea* and *igapó* floodplain forests (with lower GIA and GIR), together with the speciose *terra firme* galling community emerge as the gall diversity apex landscape among all biogeographic regions already investigated. Contrary to expectation, our results also support the “harsh environment hypothesis”, and unveil the Amazonian upper canopy as similar to Mediterranean vegetation habitats, hygrothermically stressed environments with leaf temperature at lethal limits and high levels of leaf sclerophylly.

## Introduction

In spite of the increasing knowledge on the spatial distribution patterns of many species worldwide, the evolutionary processes and ecological mechanisms shaping them remain poorly known, due to geographic and physiological limitations. At least in some rare cases advances have been made over the last decades, such as in the interactions between galling insects and their host plants. The studies on galling insects have been successfully used as tools to assess the main factors affecting the distribution patterns and diversity of insects (e.g., [1]–[7]).

Peak diversity of galling insects has been widely recorded at latitudes between 24°–45° N/S, or equivalent altitude. So far, sampling sites in Arizona (USA), Australia, Israel, South Africa, and Minas Gerais (Brazil) presented the greatest richness of galling insects [3], [8]. Furthermore, increases in the richness of these insects with decreasing site altitude were reported in Arizona (USA), Indonesia, and Minas Gerais (Brazil) [1], [8]–[11]. A relatively large number of studies reassert the strong relationship between galling insect diversity and hydric, thermal, and nutritional stresses in some habitats, resulting in an overall pattern of a greater number of galling species [3], [12]–[16]. This endophytic insect fauna would benefit from the protection against desiccation, sunlight radiation, and natural enemies (free-living herbivores, predators, fungi, other pathogens) in these habitats, and by nutritive tissues provided by gall structure [8]. Hence, this pattern (higher galling diversity in stressed environments) was consistently demonstrated in the understory of Neotropical scleromorphic vegetation (response to low nutrient levels [17]), xeromorphic vegetation (response to low water levels [17]) in the northern hemisphere, and non-scleromorphic vegetation in the mesic latitudes. However, few studies have been developed in the Neotropical vegetation with non- scleromorphic physiognomy ([18], but see [19], [20]).

Studies evaluating galling insect diversity have usually sampled shrubs and lower stature trees; as a consequence these studies encompass the canopy and understory of xeric/scleromorphic vegetation while taller trees in rainforests have been investigated less frequently [20], [21]. Except in a few studies, galling insect richness and abundance associated with the canopy of mesic/non-scleromorphic vegetation remained underestimated for some time. A pioneer study from 1998 found that the understory of moist forests was richer in galling species, compared to tree canopy of a secondary forest near Porto Velho (RO, Brazil), and in Panama [3]. With the increased availability and use of sampling techniques such as canopy cranes, access limitations were overcome, and a higher richness of galling insects in the canopy was found in the tropical forests of Panama, when compared to its understory [22]–[24]. These results indicate that the harsh conditions, including host leaf sclerophylly, observed in the Mediterranean types of vegetation and in the canopy of tropical rain forests may also favor galling. Leaf sclerophylly occurs in several vegetation types, along a broad climatic and geographic range; it includes hard, thick and tough leaves, and can be expressed by the ratio between crude fiber to crude protein content. This trait has been proposed as an adaptation to water and nutrient limitation, as well as protection against herbivore damage [25].

The assortment of vegetation found in the Amazon region is enormous as well as variation within habitats (e.g., [26], [27]). Water level changes dramatically in flooded forests [28], while different nutrient concentration and soil physical properties engender the development of endless strategies and adaptations by the flora, which is reflected in the associated community [29]–[31]. *Várzea* forests, for instance, are forests seasonally flooded by water that carries nutrient-rich sediments; consequently they have fertile soils and a diverse flora and fauna. The oligotrophic *igapó* forests are sluiced with acid and nutrient poor water, resulting in soils with nutrient scarcity, and lower numbers of plant and animal species than *várzea* [28]–[30], [32], depending on the taxa and on the geographical area. In both flooded forests, while some tree species lose their leaves during the flooding peak [33], and flush new leaves when water levels diminish, other species retain their green leaves throughout the flooding season [28]. Another vegetation type, the *terra firme* forest, is characterized by lack of flooding and high species diversity with plants growing in dry and poor soils in upland terrain of the Amazonian basin [27], [34]. Diversity in *terra firme* forest is maintained through several plant adaptations to ensure economic and efficient nutrient cycling. Main adjustments by this upland flora include a dense and superficial mesh of fine roots, thereby increasing the nutrient absorption area, and association with arbuscular mycorrhizal fungi [35].

A gradient in soil fertility exists naturally in the Amazonian rain forest: the *várzea* soils represent the most fertile ones followed by the upland *terra firme* and *igapó* floodplain [30]. According to the hypothesis of nutritional stress [1], [2], [36], it is expected that plants located in these different landscapes would be exposed to a large array or levels of physiological stress, which would result in differential richness and abundance of galling insects. In this study, we hypothesize that upland *terra firme* and *igapó* forests would present greater diversity of galling insects than *várzea* forests. Considering that *terra firme* has an extraordinarily diverse flora, with average richness of ca. 280 tree species (≥10 cm dbh) per hectare [34], we also expect that this landscape would present the highest galling insect richness as an outcome of the wide availability of ecological niches and resources [11], [37], [38]. Therefore, richness of galling insects would decrease from upland *terra firme*> *igapó*> *várzea* forests.

Thus, the goals of this study were to: (i) compare the richness, abundance and composition of galling insects between three types of Amazonian forests, located in soils with different hydric and nutritional status, and (ii) evaluate the relationship between galling insect and tree richness in such discrete landscapes. Furthermore, we contextualize our results in the scenario observed in the literature and attempt to detect and discuss possible mechanisms involved in the association of galling insect richness with harsh and stressed habitats.

## Materials and Methods

### Ethics Statement

Collection and transport of plant and galling insect specimens involved in this research were authorized by the Brazilian Institute of Environment and Renewable Natural Resources (IBAMA-AM, Permit numbers 23/2004; 04-DITEC/2006; 054NUFAS/2004; 31/2006-NUFAS).

### Study Area

**Figure 1:**
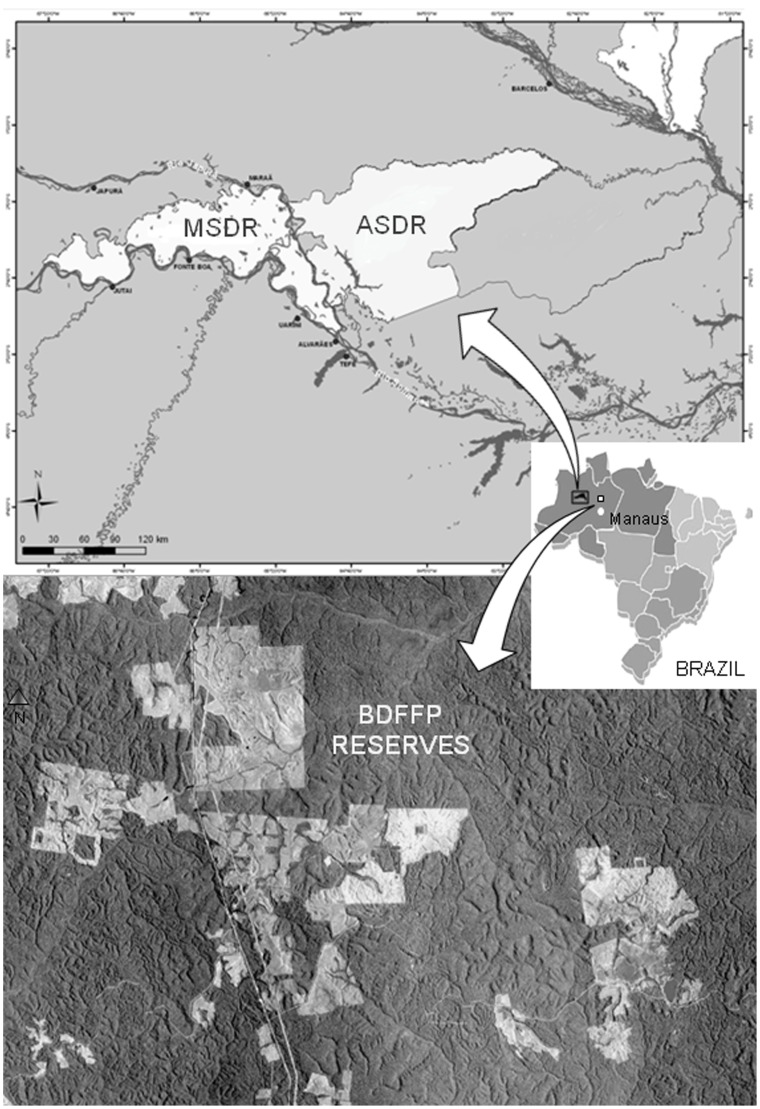
Study area at the BDFFP, MSDR, and ASDR reserves, Central Amazonia, Brazil. Map sources: http://www.pdbff.inpa.gov.br/area3p.htm (Biological Dynamics of Forest Fragments Project - BDFFP); http://www.mamiraua.org.br/downloads/mapas [Mamirauá (MSDR) and Amanã (ASDR) Sustainable Development Reserves].

The study was conducted in the reserves of the Biological Dynamics of Forest Fragments Project (BDFFP, 2°30′S; 60°W), located about 70 km north of Manaus, in Mamirauá Sustainable Development Reserve (MSDR, 2°51′S, 64°55′W), and Amanã Sustainable Development Reserve (ASDR, 2°26″S; 64°47′W) near the municipality of Tefé, Amazonia, Brazil. Samplings were done between May 2004 and December 2005. BDFFP reserves consist of *terra firme* forests exclusively, which are never flooded (Fig. 1). MSDR is situated in the interfluvial land between the Japurá and Solimões Rivers; a large extension of this reserve is subjected to flooding, being characterized as a floodplain. Placed in the middle of Solimões River, ASDR comprises *várzea* habitats, but due to the influence of the Rio Negro basin, some habitats are characterized as *igapó*. Along with the Jaú National Park, MSDR and ASDR form the largest biological corridor of preserved tropical rainforest in the world. Overall, 56 sampling points were established in the BDFFP reserves (28 sites), in MSDR (8 sites) and in ASDR (20 sites); all reserves are located within Amazonas State, Brazil. These three forest types also differ in vegetation structure and composition. The upper canopy of *terra firme* reaches 30–37 m, some emergent trees grow to 45–50 m, and the forest is dominated by Sapotaceae, Lecythidaceae, and Burseraceae tree species [34]. Euphorbiaceae is the most important botanical family in the *várzea* and *igapó* forests [30]. *Várzea* forest contains a wide range of canopy heights, depending on the forest zonation: trees can be 15–35 m or 35–45 m tall, and a few emergents which reach 58 m [39], [40]. In future publications, we will describe host and non-host tree species, genera and families of MSDR, ASDR, and BDFFP reserves.

### Galling Insect and Host Plant Sampling

Each sample point consisted of eight plots of 5×20 m, which were 20 m apart from each other (total area of 800 m^2^). The set of plots was established in the forest understory with a measuring tape and then visually projected on the canopy. The sample area was delimited, and the individual tree crowns were distinguished in the upper canopy (characterized by no shading from other trees and positioned at the air-canopy interface). Then, a haphazard sampling was performed in each individual tree crown, by clipping 10 terminal units of the plant [20], [41] using the peconha climbing technique (known as the “foot-belt” [42]) and a telescoping aluminum pole (10 m). In our case, terminal unit length ranged from 30–50 cm, and it encompassed branches, stems, leaves, flowers, and fruits (the last two structures, if they were present). In the field, insect gall morphotypes were recorded as well as their abundances for each tree individual. Gall morphotypes were characterized by external morphology, shape, color, trichomes, single or grouped occurrence, and the host plant organ attacked (see [1]). Due to high specificity in the relationship between galling insects and their host plants, each gall morphotype was considered a species of galling insect (see [43] for a review). After this, galling insect richness (GIR) and galling insect abundance (GIA) were estimated from the number of galling species and number of individual galls for each morphotype, respectively.

### Data analysis

Galling insect richness (GIR) and galling insect abundance (GIA) were considered response variables (separately) and forest type and number of tree species sampled (TSS: unattacked and attacked trees) as predictors, as well the interaction between them. We built GLM (generalized linear models) assuming Poisson error distribution, and compared them with Chi squared tests, in order to deal with overdispersion observed in the data [44]. To verify possible effects on GIR and GIA, we used two approaches recommended by Crawley [44]: (i) ANCOVA-GLM model simplification (full model) and (ii) ANOVA-GLM model contrast, to check the effects of the forest type levels in the explanatory power of a model. In the first one, a full model included forest type categories (*várzea*, *igapó* and *terra firme*), TSS, and the interaction term. The models were manually updated by excluding a combination of explanatory variables. After this, initial and final models were compared by Chi squared tests. Model contrast (second approach) was employed when pairs of factor levels (categorical variable) presented similar parameter values (in our case, mean). Forest type levels (*várzea* + *igapó* categories) were concatenated, and model parameters were compared with *z*-test. The variation in the gall morphospecies composition (presence/absence) among forest types was evaluated with Jaccard dissimilarity index, running vegan package (version 2.0–10). All analyses were done in the R software program [44].

### Amazonian GI Diversity x Literature Patterns

Two variables were used to compare results obtained in this study and patterns of distribution and diversity of galling insects recorded in the literature: (i) galling insect richness (GIR) per sample point, and (ii) the ratio GIR/TSS in which the first variable is divided by the number of tree species sampled (host plants and non-hosts; TSS). In this study, the ratio GIR/TSS values of each landscape were computed as averages per sample point (*terra firme* forest: n = 14; *igapó* forest: n = 14; *várzea* forest: n = 14).

**Figure 2:**
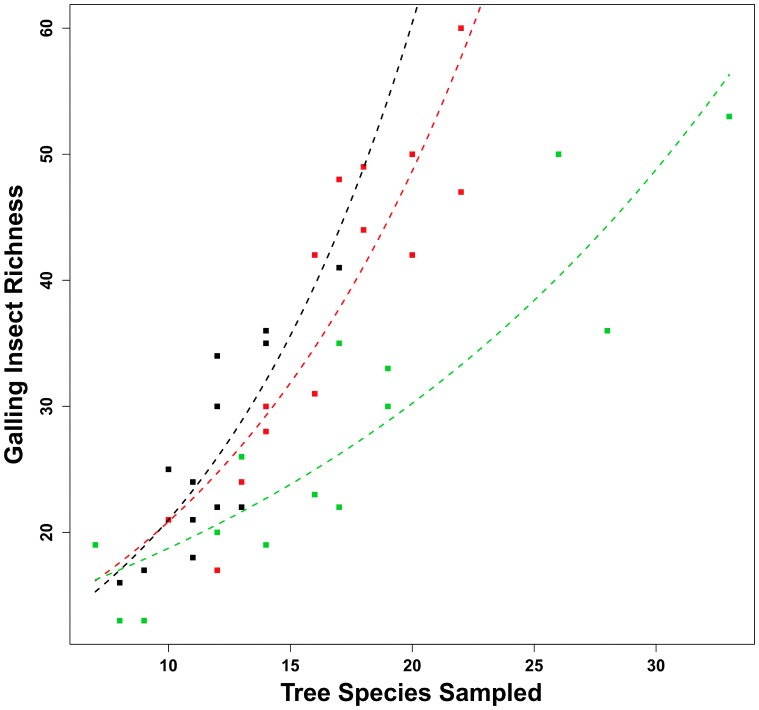
Relationship between galling insect richness (GIR) and number of tree species sampled (TSS) in the *terra firme* (red curve and symbols), *várzea* (green curve and symbols), and *igapó* (black curve and symbols) forests at Amazon. GIR variation explained by TSS differed among levels of forest type.

GIR values obtained in this study were also plotted onto Fig. 2 (page 586) of the study published by Price *et al.* [3], where GIR's from various biogeographic regions on an extensive latitudinal gradient were combined. This study emphasized vegetation traits (scleromorphic and non- scleromorphic) and habitat types (xeric, away from water bodies, or mesic, near water bodies). GIR comparisons were visually made. Some studies in this review had adopted the architectural census sampling, which included the intensive investigation of gall insects on 45 trees, 100 shrubs, and 1000 herbs. However, 45 trees and 100 shrubs were only collected in a few studies (see [45]), while most studies employed the 60-minute census, shown to be equivalent to the architectural census [3].

The sampling method employed here (800 m^2^ sampling area) does not encompass a record of shrubs and herbaceous plants; nevertheless, number of trees sampled at each site can be used in the data comparisons. Almost all of *várzea*, *igapó,* and *terra firme* sites were represented by fewer than 45 individual trees (the number proposed as sufficient to reach an asymptote in species richness [1]), with the exception of one sampling site in the *várzea* forest (Sampled trees  = 48 individuals; GIR = 53 morphospecies). In addition, Fernandes and Price [1] observed that 90% of galling species were collected, on average, after sampling 26 individual trees. In this study, a range of ten to 25 tree individuals were found in 38 sites, while 27, 31, 34, and 48 trees were sampled in the other four remaining sites.

GIR/TSS ratios estimated for *terra firme*, *igapó*, and *várzea* forests were compared to the two highest and two lowest ratio values compiled by Espírito-Santo and Fernandes ([4], but see [14], [46]–[48]). In addition, values were compared to the GIR/TSS values found for tropical forests of Panama [49]. However, sampling methods used in these studies varied widely and galling diversity can be affected by plant community composition [18] and plant richness [11], [38]. We tentatively minimize the GIR over- and under-estimation for a given location/site using the GIR/TSS ratio once sampling effects can be controlled by the number of tree species sampled (host plants and non-hosts).

## Results

Overall, we examined 1,091 tree individuals, which were identified as 491 species belonging to 49 botanical families. Out of this total, 89.6% of the trees (978 individuals) were attacked by galling insects, comprising 445 host tree species and 46 non-host tree species. In addition, 141,244 galls induced by 1,150 morphospecies of galling insects were recorded. However, only part of the dataset was included in our statistical analysis in order to balance sampling efforts at different forest types (S1 Table). Higher abundance and richness of galling insects were found in the *terra firme* forest, followed by *várz*ea and *igapó* habitats (Table 1). However, *igapó* forest presented more galling species *per* tree species sampled (GIR/TSS) than *várz*ea forest (Table 2).

**Table 1:** Number of individuals and species of host plants; galling insect richness (GIR), abundance (GIA), and dissimilarity among the *terra firme*, *várzea,* and *igapó* forests in Amazon, Brazil.

	Host Plant			Galling Insect		Jaccard Dissimilarity to	
Forest Type	N*	Individuals	Species	GIR	GIA	*Terra Firme*	*Várzea*
*Terra Firme*	14	242	165	428	52,055		
*Várzea*	14	246	127	297	26,244	0.9874	
*Igapó*	14	229	100	235	23,994	0.9608	0.9098

*N: number of sampling sites in each landscape.

**Table 2:** Ratios between galling insect richness and number of tree species sampled (GIR/TSS) in the understory (U) and canopy (C) of different vegetation types in several biogeographic regions.

Vegetation type	Locality/Country	Habitat	GIR/TSS	Sampling Area (m^2^)
Tropical Savanna [4], [14]	NATT/Australia	U+C	0.50	10,000
Fynbos[4], [46]	Cape Floristic Province/South Africa	-	0.48	-
Various [4], [47]	Various/Taiwan	-	0.05	-
Montane-Desert gradient [4], [48]	Big Bend National Park/USA	-	0.14	2,000
Dry Tropical Forest [49]	Parque Natural Metropolitano/Panama	C	0.64	8,100
Dry Tropical Forest [49]	Parque Natural Metropolitano/Panama	U	0.18	-
Tropical Rainforest [49]	Fuerte Sherman/Panama	C	0.66	9,000
Tropical Rainforest [49]	Fuerte Sherman/Panama	U	0.31	-
Tropical Rainforest/Terra firme*	BDFFP/Brazil	C	2.25	11,200
Tropical Rainforest/Várzea *	MDSR, ADSR/Brazil	C	1.69	11,200
Tropical Rainforest/Igapó *	ADSR/Brazil	C	2.19	11,200

*Our Study.

### Galling Insect Richness x Tree Species x Forest Type

Our initial (maximal) model was highly significant (d.f. = 36,41; P<0.001) and explained 83% of variability observed in the galling insect richness (GIR). The predictor variables were forest type (categorical: *terra firme*, *várzea*, and *igapó*), TSS (the number of tree species sampled), and interaction between them. A significant variation was also observed in GIR when we removed the interaction and compared with null model (R^2^ = 0.77; d.f. = 38,41; P<0.001). However, there was a statistical difference between the two models (d.f. = 38,36; P<0.01), indicating the importance of the interaction as an explanatory term (Fig. 2), and hence, it was retained in the analysis. In concern to the individual effects of forest types (*terra firme*, *várzea*, and *igapó*) on the values of gall richness, level concatenation (flooded forests: *várzea* + *igapó*) produced a significant model (d.f. = 38,41; P<0.001, R^2^ = 0.74), but also significantly different from our initial model. Again, there was no justification to maintain a final model based on this simplification approach. Although our initial model presented few significant parameters, higher *z* values were observed on the number of tree species sampled (TSS) in igapó and igapó forest type, and the interaction between *várzea* category and its TSS.

### Galling Insect Abundance x Tree Species x Forest Type

**Figure 3:**
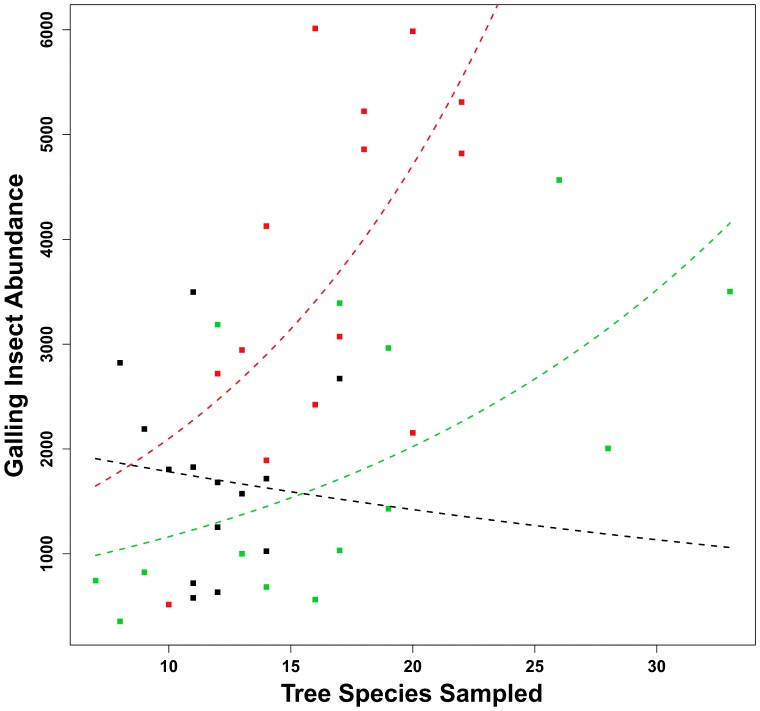
Relationship between galling insect abundance (GIA) and number of tree species sampled (TSS) in the *terra firme* (red curve and symbols), *várzea* (green curve and symbols), and *igapó* (black curve and symbols) forests at Amazon. All parameters were significant in our initial model, in spite of its insufficient predictive power.

A significant amount of variability in GIA was not explained by the ANCOVA-GLM (d.f. = 36,41; P<0.001; R^2^ = 0.52), but all parameters (and their interactions) were highly significant in the model (Fig. 3). Simplification approaches were, however, employed in order to access explanatory power of the final models. Both the removal of TSS*forest type interaction and level concatenation in forest type resulted in lower values of R^2^ (both models: 49%; d.f. = 38,41; P<0.001).

### Gall Morphospecies Composition

**Figure 4:**
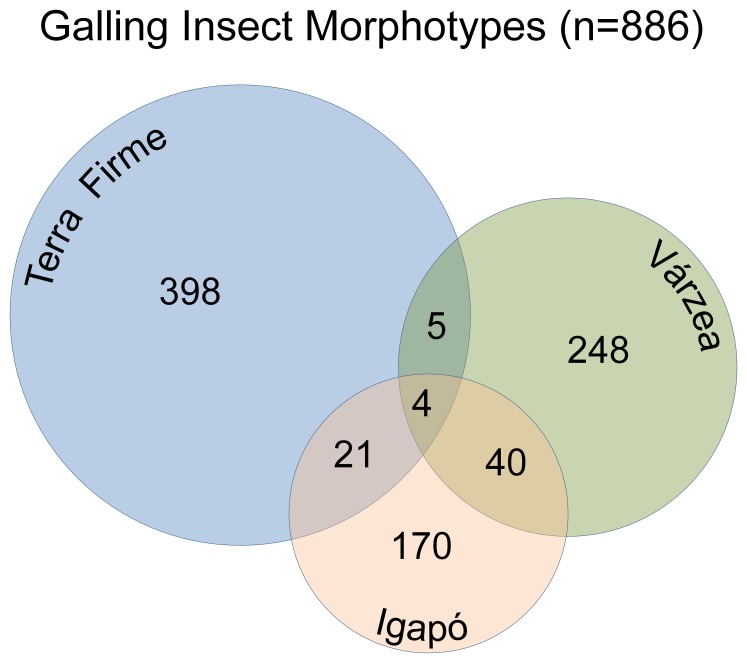
A Venn diagram representing the number of galling morphospecies exclusive and common to the *terra firme* (blue), *várzea* (green), and *igapó* (orange) forests, Central Amazonia, Brazil. Presence/absence data revealed great dissimilarity among Amazonian forest types, even considering the flooded forests.

Overall, 886 morphospecies of gall-forming insects were discovered in the fourteen-site sample at each Amazonian forest type. Only a minor proportion of galling insects (∼8%, n = 70) was shared among *terra firme*, *várzea* and *igapó* forests (Fig. 4). Forty-four morphospecies were common to the flooded forests, *várzea* and i*gapó*. Gall morphospecies composition differed between pairs of forest types and higher values of Jaccard dissimilarities were observed (Table 1).

### Global Patterns in Galling Insect Diversity

**Figure 5:**
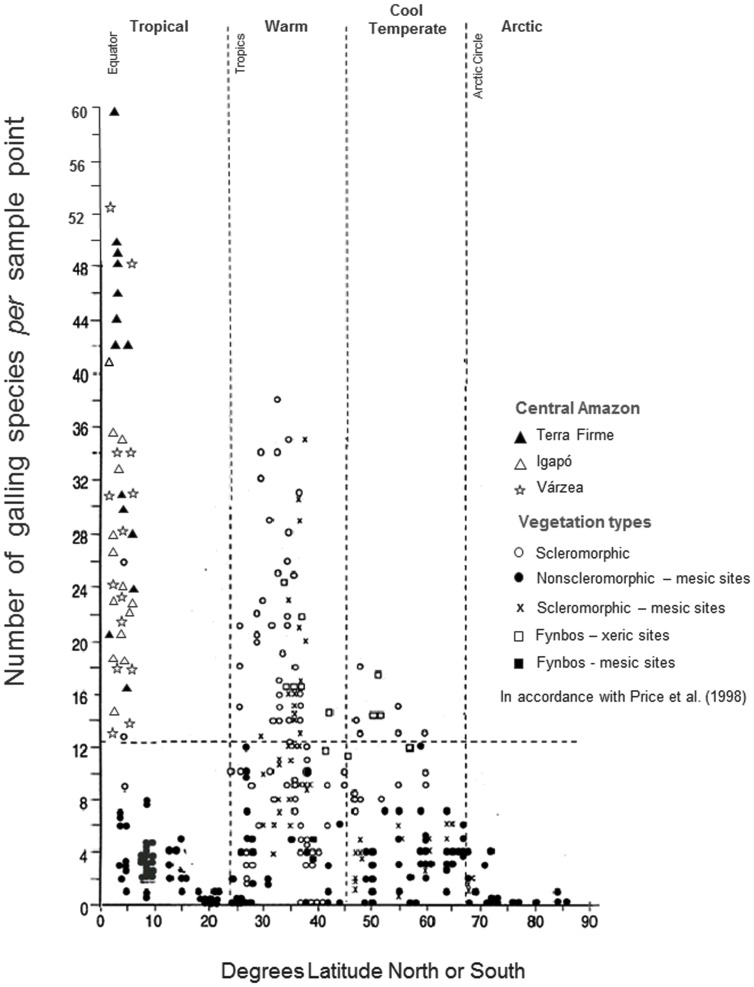
GIR in Central Amazon sites and values from other vegetations types from Brazil and world. The figure was re-drawn from Fig. 2, page 586, work of Price *et al.* [3]. GIR (galling insect richness) is equivalent to the number of galling species *per* sample point.

The original studies of Fernandes and Price [1], [2] established the first trends of habitat-related patterns in galling insect distribution. Using a different sampling protocol, the highest number of galling insects recorded by Fernandes and Price [9] reached 46 morphospecies. Fig. 5 illustrates the present Amazonian galling richness (42 sites) in comparison with the values for other vegetation of Brazil and the world, compiled by Price *et al.* [3] and Price [45]. Absolute GIR fluctuated greatly among sites and forest types in central Amazon rainforests. GIR ranged from 60 galling insect morphospecies, found in a sample point of upland *terra firme* (BDFFP continuous forest) to 13 morphospecies, collected in a *várzea* ASDR site, with the same 800 m^2^ sampling area. Visual comparison highlighted that GIR highest value - 60 morphospecies - was observed in an Amazonian site, where 22 tree individuals were examined.

GIR/TSS ratios were compared among *terra firme*, *igapó*, and *várzea* forests and contrasted with the values reported in the literature (Table 2). The lowest value of this ratio in the Amazonian forest was found in the *várzea* forest. However, tropical rainforest ratios were, at least, 2.5× higher than the results reported in the literature [49]. This also indicates the highest galling insect diversity ever recorded for any biogeographic realm so far.

## Discussion

The sampling of galls at the upper canopy of three Amazonian rain forest types indicated that both galling insect richness and abundance were higher in upland *terra firme* forests located on poor and strongly leached soils and lower in the floodplain forests. Given that *igapó* forests had been characterized as nutrient-poor habitats by some authors [50], [51], we would expect a high galling diversity in such environments (see [1], [9]). In contrast, lower absolute values of GIR and GIA were found in the *igapó* forests; the reasons for this await further investigations based on plant physiology, chemical and mechanical defenses against herbivores, and relationships between flood tolerance and herbivory.

Disregard with the explanatory power of models (amount of variability in response variable explained by the predictor variables), GIR and GIA were highly influenced by the number of trees species sampled (host and non-host plants) in each forest type (interaction term). Our data support the hypothesis that the estimated parameters to richness and abundance of galling insects are intimately dependent on host plant diversity (S2 Table), and vegetation community where they are located, as observed in the *igapó* forest. In addition, we unveiled some environmental filters shaping galling species patterns in the Amazonian forest ecosystems - vertical gradient in leaf traits, flooding, species composition, soil/water nutrients, and super host occurrence.

Impoverished floras have been consistently recorded in the *igapó* forests in comparison to *várzea* and upland *terra firme* forests (e.g., [26], [30], [32]). The arthropod fauna (oribatid mites and spiders) seems to be more speciose in *igapó* than *várzea* [52], [53]. We also found that *igapó* forest had a greater number of galling species per plant species than *várzea*, in spite of their floristic similarity. GIR/TSS ratios revealed that *igapó* plants accumulated a larger number of galling species, similar to upland *terra firme* trees. Such similarity between *terra firme* and *igapó* could be explained by the low nutritional status of their soils, given that these landscapes are very distinct in regard to hydrological features. Haugaasen and Peres [30] verified that *várzea* soils of the lower Purús River were richer in nutrients, while *terra firme* forests and *igapó* had no significant differences regarding their nutritional status (Ca, Mg, Al, Zn and Mn, excepting phosphorus in the *igapó*). Therefore, apart from the influence of number of plant species in a given location or type of vegetation, this study supports the nutritional stress hypothesis [1], [2], [36], which predicts a greater GIR in environments whose plants are subject to nutrient limitation.

*Igapó* flooded forests have additional characteristics that make them prone to a high diversity of galling insects. Similarities based on taxonomic and biogeographic traits were found between *igapó* flora and oligotrophic habitats in the Amazon savannas, “caatingas”, and white sand savanna, which are located on poor soils [54], [55]. Moreover, some areas of *igapó* vegetation experience desert-like conditions in the dry season and its plants exhibit xeromorphic adaptations such as sclerophyllous leaves [26]. On the other hand, *várzea* floodplain vegetation is more related to the vegetation growing on fertile habitats from upland *terra firme* forest [56]. Nutrient-poor soils and large water table fluctuations have been argued to be the main explanation of enhancement of leaf construction costs, and a thickness and sclerophylly index in microhabitats of mixed forests of Venezuela [57]. In lowland tropical forest of Panamá, significant higher leaf sclerophylly was found on the higher strata of rainforest, and insect gall richness was positively affected by sclerophylly [22]. The alterations in leaf traits (area, mass/area, thickness, anatomy) have been also related to an ontogenetic transition between sapling/emergent tree life stages, besides the harsh conditions at the upper canopy [58].

A canopy measurement study from four towers situated in *terra firme* forests from Central Amazon, Brazil, evaluated the effects of leaf temperature variation on respiration and photosynthesis. It revealed that some canopy leaves reach temperatures very close to their lethal limit [59]. The author also found that the temperature of sunlit leaves exceeded air temperature by 6°C, on average, in both rainy and dry seasons, and reached values 10°C above air temperature. Surprisingly, values for canopy leaf temperatures surpass 45°C, indicating that upper canopy leaves experience a habitat under strong thermal stress [59], [60]. Morphological and physiological alterations in the upper canopy leaves help them tolerate such stress; lower values of specific leaf area indicate such leaves have larger amounts of leaf mass per area unit [61]. Of 21 tree individuals identified at the species level by Tribuzy [59], fourteen individuals (66.7%) belonged to tree species also sampled in our study. These plant species hosted 40 galling insect morphospecies and accounted for 7.4% of total gall abundance (10,467 galls). Considering a vertical profile of leaf traits, leaves which grow in full sunlight were hard, with lower specific leaf area (SLA), and a higher sclerophylly index than shaded leaves in the Brazilian Central Amazon [62]–[64]; the specific leaf area has also been used to characterize the sclerophyllous leaves in several vegetation types [65], [66].

However, evidence suggests that flooding may represent a strong regional selective pressure on these floodplain forest systems. Leaves of Amazonian floodplain trees present several traits which help with the scanty water supplies to the crowns, during the flooding period. On average, 5–33% higher values of specific leaf mass (another indirect measure of sclerophylly) were found during the flooding period than in the non-flooded months [67], [68]. For instance, a flood-adapted species, *Calophyllum brasiliense* (Clusiaceae) hosts five leaf and stem galling species (*Lopesia caulinaris*, *L.conspicua*, *L. elliptica*, *L. linearis* and *Contarinia gemmae*) [69]. On this species, Ribeiro *et al.* [70] reported a high frequency of galling attack on flooded individuals compared to non-flooded ones. The abundance of galls and the number of successfully emerged adults per leaf was also higher on plants subjected to flooding [70]. We conclude that selection for sclerophyllous foliage could be an adaptive mechanism (sunlight, flooding) which has favored the galling insect fauna.

Galling insect abundance was also higher in the *terra firme* landscape where we also recorded larger numbers of insect galls per host plant. Tree hosts from *igapó* and *várzea* supported similar but lower values of galling insect abundance. However, which factors modulate the number of galls per insect species in the Amazonian forests deserve more attention in future studies, since interactions with other organisms could affect directly or indirectly the abundance of gall-forming insects [71].

High values of GIR/TSS ratios obtained in this study could be explained by the small number of plant species not attacked by galling insects (in our study, only tall trees), and the large number of “superhost” species; i.e, plant species attacked by a large number of galling species [1], [72], [73]. In the *várzea* forest we observed the highest number of tree species not attacked by galling insects (45 tree species). *Terra firme* and *igapó* forests presented much smaller numbers of non-host trees, 26 and 19 tree species, respectively. In our study, *Protium altsonii* Sandw., *P. tenuifolium* (Engl.) Engl. (Burseraceae), and *Licania micrantha* Miq. (Chrysobalanaceae) were the host tree species which presented higher number of gall morphospecies (ten morphotypes per tree species); the first two were collected only in *terra firme* sites while the third one was sampled in *terra firme* and *igapó* forests.

Knowledge on plant diversity, as well as floristic composition of a site, have been assigned as main relevant factors in the analysis of distribution patterns of galling insect communities [38], [74]. Veldtman and McGeoch [18] found that the floristic composition was a major factor in local GIR through the presence of “superhosts”. This pattern was also observed for free-living insect herbivores associated with the forest canopy of Laurisilva, Azores [75]. Out of 129 herbivorous insect species found, 65 species were sampled on *Juniperus brevifolia*, and 53 species on the *Erica azorica*. In this study, Fabaceae accumulated 222 galling morphospecies (mainly on the genus *Inga*; nineteen *Inga* species supported 69 galling morphotypes), followed by the families Sapotaceae and Lecythidaceae, with 159 and 106 galling insect morphospecies, respectively. Carvalho-Fernandes [76] also found 95 galling morphospecies associated with 15 *Protium* species (Burseraceae) in a *terra firme* forest, near Manaus (Amazonas, Brazil). Further analysis will provide inferences about the role of each host plant species in galling diversity patterns observed in the canopy of *terra firme*, *várzea,* and *igapó* forests.

To elaborate and refine the explanatory hypotheses related to galling insect distribution patterns and galling insect diversity, it is necessary to investigate other systems, and preferably, vegetation types which contrast with the scenarios where the main hypotheses have been formulated and corroborated. By doing so, we reported larger gall richness than those recorded to date. GIR/TSS ratio in the *várzea* forest was approximately 2.5 times larger than the largest value of this ratio ever recorded in the literature (canopy of tropical forest, Panama, [49]). The clear difference between ratio values indicates that even the GIR poorest site in Amazonian vegetation is among the habitats with the greatest diversity of galling species of all biogeographic regions already investigated.

**Figure 6:**
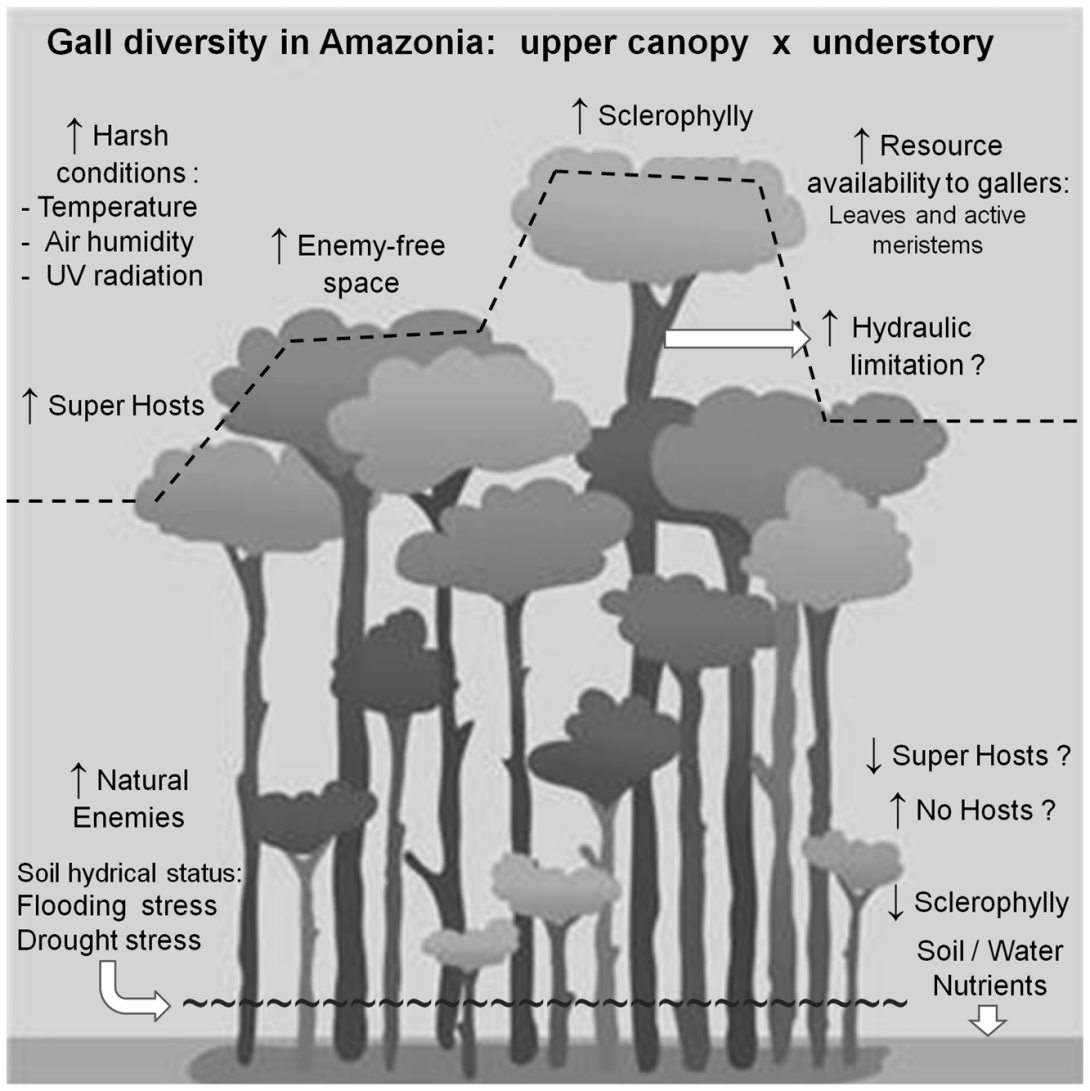
Mechanisms shaping the distribution patterns of galling insects in the Amazonian forests. Down arrow icon means lower value/level; up arrow icon, higher ones in the comparison between canopy and understory.

Despite that galling insect diversity peaks having been reported exclusively for scleromorphic vegetation, our results strongly support the hypothesis of Fernandes and Price [1], [9], Price *et al.* [3] and Ribeiro [21], and unveil the Amazonian upper canopy as a hygrothermically stressed habitat, which possess high levels of sclerophylly, compared to the understory habitat [62]–[64], a non- scleromorphic humid forests of central Amazonia (Fig. 6). Ribeiro and Basset [22] showed the importance of leaf sclerophylly to galler oviposition preferences in the upper canopy, resulting in an enemy-free space (death by fungi and free-feeding herbivores). Besides, the effects of hydraulic stress in large tropical trees and the number of active meristems (higher in canopy than understory) on the galling insect richness remains to be tested (Fig. 6). In spite of being in a humid domain, the canopy of the Amazonian Equatorial forest might most resemble a savanna or Mediterranean type environment with high temperatures and UV radiation where galling herbivores flourish.

## Supporting Information

S1 Table **Sampling points in the Amazonian forest types, the richness and abundance of galling insects, host plants, and geographical location.**
(PDF)

S2 Table **Host plant list of the Amazonian gall-forming insects.**
(PDF)

## References

[1] Fernandes GW, Price PW (1988) Biogeographical gradients in galling species richness: test of hypotheses. Oecologia 76:161–167. 28312192 10.1007/BF00379948

[2] Fernandes GW, Price PW (1992) The adaptive significance of insect gall distribution: survivorship of species in xeric and mesic habitats. Oecologia 90:14–20. 28312265 10.1007/BF00317803

[3] Price PW, Fernandes GW, Lara ACF, Brawn J, Barrios H, et al (1998) Global patterns in local number of insect galling species. J Biogeogr 25:581–591.

[4] Espírito-Santo MM, Fernandes GW (2007) How many species of gall-inducing insects are there on Earth, and where are they? Ann Entomol Soc Am 100:95–99.

[5] Tooker JF, Rohr JR, Abrahamson WG, De Moraes CM (2008) Gall insects can evade and alter indirect plant defenses. New Phytol 178:657–671. 18331430 10.1111/j.1469-8137.2008.02392.x

[6] Medianero E, Ibáñez A, Nieves-Aldrey JL (2010) Importance of beta diversity in local gall-inducing arthropods communities. Neotrop Entomol 39:365–370. 20676509 10.1590/s1519-566x2010000300009

[7] Sullivan LL, Johnson BL, Brudvig LA, Haddad NM (2011) Can dispersal mode predict corridor effects on plant parasites? Ecology 92:1559–1564. 21905422 10.1890/10-1116.1

[8] Fernandes GW, Gonçalves-Alvim SJ, Carneiro MAA (2005) Habitat-driven effects on the diversity of gall-inducing insects in the Brazilian cerrado. In: Raman A, Schaefer CW, Withers TM, editors.Biology, ecology, and evolution of gall-inducing arthropods.Enfield, USA: Science Publishers. pp. 693–708.

[9] Fernandes GW, Price PW (1991) Comparisons of tropical and temperate galling species richness: the role of environmental harshness and plant nutrient status. In: Price PW, Lewinsohn TM, Fernandes GW, Benson WW, editors.Plant-animal interactions: evolutionary ecology in tropical and temperate regions.New York: John Wiley. pp. 91–116.

[10] Fernandes GW, Lara ACF (1993) Diversity of Indonesian gall-forming herbivores along altitudinal gradients. Biodivers Letters 1:186–192.

[11] Lara ACF, Fernandes GW, Gonçalves-Alvim SJ (2002) Tests of hypotheses on patterns of gall distribution along an altitudinal gradient. Trop Zool 15:219–232.

[12] Lara ACF, Fernandes GW (1996) The highest diversity of galling insects: Serra do Cipó, Brazil. Biodivers Letters 3:111–114.

[13] Fernandes GW, Araújo RC, Araújo SC, Lombardi JA, Paula AS, et al (1997) Insect galls from Jequitinhonha Valley, Minas Gerais, Brazil. Naturalia 22:221–224.

[14] Blanche KR (2000) Diversity of insect-induced galls along a temperature-rainfall gradient in the tropical savannah region of the northern territory, Australia. Aust Ecol 25:311–318.

[15] Cuevas-Reyes P, Quesada M, Siebe C, Oyama K (2004) Spatial patterns of herbivory by gall-forming insects: a test of the soil fertility hypothesis in a Mexican tropical dry forest. Oikos 107:181–189.

[16] Ribeiro SP, Silva Jr. MB, Tagliati MC, Chavana-Bryant C (2011) Vegetation traits and herbivory distribution in an Australian subtropical forest. Mem Queensl Mus - Nature 55:481–493.

[17] Hill RS (1998) Fossil evidence for the onset of xeromorphy and scleromorphy in Australian Proteaceae. Aust Syst Bot 11:391–400.

[18] Veldtman R, McGeoch MA (2003) Gall-forming insect species richness along a non-scleromorphic vegetation rainfall gradient in South Africa: The importance of plant community composition. Aust Ecol 28:1–13.

[19] Yukawa J, Tokuda M, Uechi N, Sato S (2001) Species richness of galling arthropods in Manaus, Amazon and surroundings of the Iguassu Falls. Esakia 41:11–15.

[20] Julião GR, Venticinque EM, Fernandes GW (2005) Richness and abundance of gall-forming insects in the Mamirauá Várzea, a flooded Amazonian forest. Uakari 1:39–42.

[21] Ribeiro SP (2003) Insect herbivores in the canopies of savannas and rainforests. In: Basset Y, Novotny V, Miller SE, Kitching RL, editors.Arthropods of Tropical Forests: spatio-temporal dynamics and resource use in the canopy.Cambridge, UK: Cambridge University Press. pp. 348–359.

[22] Ribeiro SP, Basset Y (2007) Gall-forming and free-feeding herbivory along vertical gradients in a lowland tropical rainforest: the importance of leaf sclerophylly. Ecography 30:663–672.

[23] Medianero E, Barrios H (2001) Riqueza de insectos cecidógenos en el dosel y sotobosque de dos zonas ecológicas en Panamá. Scientia (Panamá) 16:17–42.

[24] Paniagua MR, Medianero E, Lewis OT (2009) Structure and vertical stratification of plant galler-parasitoid food webs in two tropical forests. Ecol Entomol 34:310–320.

[25] Turner IM (1994) Sclerophylly: primarily protective? Funct Ecol 8:669–675.

[26] Prance GT (1979) Notes on the vegetation of Amazonia III. The terminology of Amazonian forest types subject to inundation. Brittonia 31:26–38.

[27] Pires JM (1985) The vegetation types of the Brazilian Amazon. In: Prance GT, Lovejoy TE, editors.Key environments: Amazonia.Oxford, UK: Pergamon Press. pp. 83–94.

[28] Junk WJ (1983) As águas da região Amazônica. In: Salati E, Junk WJ, Shubart HOR, Oliveira A, editors.Amazônia: desenvolvimento, integração e ecologia.Brazil: Editora Brasiliense/CNPq. pp.45–100.

[29] Junk WJ (1989) Flood tolerance and tree distribution in central Amazonian floodplains. In: Nielsen LB, Nielsen IC, Balslev H, editors.Tropical forests: botanical dynamics, speciation and diversity.London: Academic Press. pp. 47–64.

[30] Haugaasen T, Peres CA (2006) Floristic, edaphic and structural characteristics of flooded and unflooded forests in the lower Rio Purús region of central Amazonia, Brazil. Acta Amaz 36:25–36.

[31] Pereira MJR, Marques JT, Palmeirim JM (2010) Vertical stratification of bat assemblages in flooded and unflooded Amazonian forests. Curr Zool 56:469–478.

[32] Junk WJ (1984) Ecology of the várzea, floodplain of Amazonian white water rivers. In: Sioli H, editor.The Amazon: Limnology and landscape ecology of a mighty tropical river and its basin.The Netherlands, Dordrecht: W. Junk Publishers. pp. 215–243.

[33] Schöngart J, Piedade MTF, Wittmann F, Junk WJ, Worbes M (2005) Wood growth patterns of Macrolobium acaciifolium (Benth.) Benth. (Fabaceae) in Amazonian black-water and white-water floodploain forests. Oecologia 145:454–461. 16025354 10.1007/s00442-005-0147-8

[34] Oliveira AA, Mori SA (1999) A central Amazonian terra firme forest. I. High tree species richness on poor soils. Biodivers conserv 8:1219–1244.

[35] Powers JS, Treseder KK, Lerdau MT (2005) Fine roots, arbuscular mycorrhizal hyphae and soil nutrients in four neotropical rain forests: patterns across large geographic distances. New Phytol 165:913–921. 15720702 10.1111/j.1469-8137.2004.01279.x

[36] Blanche KR, Westoby M (1995) Gall-forming insect diversity is linked to soil fertility via host plant taxon. Ecology 76:2334–2337.

[37] Southwood TRE (1960) The abundance of Hawaiian trees and the number of their associated insect species. Proc Hawaiian Entomol Soc 17:229–303.

[38] Fernandes GW (1992) Plant historical and biogeographical effects on insular gall-forming species richness. Global Ecol Biogeogr Lett 2:71–74.

[39] Wittmann F, Anhuf D, Junk WJ (2002) Tree species distribution and community structure of Central Amazonian várzea forests by remote sensing techniques. J Trop Ecol 18:805–820.

[40] Wittmann F, Junk WJ, Piedade MTF (2004) The várzea forests in Amazonia: flooding and the highly dynamic geomorphology interact with natural forest succession. Forest Ecol Manag 196:199–212.

[41] Bell AD, Bell A, Dines TD (1999) Branch construction and bud defense status at canopy surface of a West African rainforest. Biol J Linn Soc 66:481–499.

[42] Malcolm JR (1991) Comparative abundances of Neotropical small mammals by trap height. J Mammal 72:188–192.

[43] Carneiro MAA, Branco CSA, Braga CED, Almada ED, Costa MBM, et al (2009) Are gall midge species (Diptera: Cecidomyiidae) host plant specialists? Rev Bras Entomol 53:365–378.

[44] Crawley MJ (2007) The R Book. West Sussex, England, John Wiley & Sons. 942p.

[45] Price PW (1991) Patterns in communities along latitudinal gradients. In: Price PW, Lewinsohn TM, Fernandes GW, Benson WW, editors.Plant-animal interactions: Evolutionary ecology in tropical and temperate regions.New York: John Wiley. pp. 51–70.

[46] Wright MG, Samways MJ (1998) Insect species richness tracking plant species richness in a diverse flora: gall-insects in the Cape Floristic Region, South Africa. Oecologia 115:427–433. 28308436 10.1007/s004420050537

[47] Yang MM, Tung GS (1998) The diversity of insect-induced galls on vascular plants in Taiwan: a preliminary report. In Csóka G, Mattson WJ, Stone GN, Price PW, editors. The Biology of Gall-Inducing Arthropods. General Technical Report NC-199, U. S. Department of Agriculture, Washington, USA. pp. 44–53.

[48] Blanche KR, Ludwig JA (2001) Species richness of gall-inducing insects and host plants along an altitudinal gradient in Big Bend National Park, Texas. Am Midl Nat 145:219–232.

[49] Medianero E, Valderrama A, Barrios H (2003) Diversidad de insectos minadores de hojas y formadores de agallas en el dosel y sotobosque del bosque tropical. Acta Zool Mex 89:153–168.

[50] Sioli H (1991) Amazônia: fundamentos da ecologia da maior região de florestas tropicais. Rio de Janeiro, Brazil: Editora Vozes. 72 p.

[51] Victoria RL, Martinelli LA, Cunha HB, Richey JE (2000) The Amazon basin and its natural cycles. In: Salati E, Absy ML, Victoria RL, editors.Amazônia: um ecossistema em transformação.Manaus: INPA, Brasília: CNPq. pp.163–214.

[52] Franklin EN, Adis J, Woas S (1997) The Oribatid Mites. In: Junk WJ, editor.The Central-Amazonian Floodplain: Ecology of a Pulsing System. Ecological Studies.Vol. 126. Berlin, Heidelberg, New York: Springer Verlag. pp. 331–349.

[53] Höfer H (1997) The spider communities. In: Junk WJ, editor.The Central-Amazonian Floodplain: Ecology of a Pulsing System. Ecological Studies.Vol. 126. Berlin, Heidelberg, New York: Springer Verlag. pp.373–383.

[54] Prance GT (1989) American tropical forests. American tropical forests. In: Lieth H, Werger MJA, editors.Tropical rain forest ecosystems - biogeographical and ecological studies.Amsterdam: Elsevier Science Publishers. p. 99–132.

[55] Kubitzki K (1989) The ecogeographical differentiation of Amazonian inundation forests. Plant Syst Evol 162:285–304.

[56] Worbes M (1997) The Forest Ecosystem of the Floodplain. In: Junk WJ, editor.The Central-Amazonian Floodplain: Ecology of a Pulsing System. Ecological Studies.Vol. 126.Berlin, Heidelberg, New York: Springer Verlag. pp. 223–266.

[57] Sobrado MA (2010) Leaf characteristics, wood anatomy and hydraulic properties in tree species from contrasting habitats within upper Rio Negro forests in the Amazon region. J Trop Ecol 26:215–226.

[58] Sanches MC, Ribeiro SP, Dalvi VC, Silva Júnior MB, Sousa HC, et al (2010) Differential leaf traits of a neotropical tree Cariniana legalis (Mart.) Kuntze (Lecythidaceae): comparing saplings and emergent trees. Trees 24:79–88.

[59] Tribuzy ES (2005) Variações da temperatura foliar do dossel e o seu efeito na taxa assimilatória de CO_2_ na Amazônia Central. Unpublished PhD thesis, Universidade de São Paulo, Brazil.

[60] Doughty CE, Goulden ML (2008) Are tropical forests near a high temperature threshold? J Geophys Res 113:G00B07 doi:10.1029/2007JG000632.

[61] Tribuzy ES, Teixeira LM, Chambers JQ, Reis TS, Trumbore S, et al. (2003) Physiological responses of tree species growing under different site conditions in the Central Amazon. In: Seventh LBA-ECO Science Team Business Meeting, Fortaleza, Brazil. LBA/INPA/NASA. p. 94

[62] Marenco RA, Gonçalves JFC, Vieira G (2001) Leaf gas exchange and carbohydrates in tropical trees differing in successional status in two light environments in central Amazonia. Tree Physiol 21:1311–1318. 11731341 10.1093/treephys/21.18.1311

[63] Gonçalves JFC, Vieira G, Marenco RA, Ferraz JBS, Santos Junior UM, et al (2005) Nutritional status and specific leaf area of mahogany and tonka bean under two light environments. Acta Amaz 35:.23–27.

[64] Camargo MAB, Marenco RA (2012) Growth, leaf and stomatal traits of crabwood (Carapa guianensis Aubl.) in central Amazonia. Revista Árvore 36:07–16.

[65] Groom PK, Lamont BB (1999) Which common indices of sclerophylly best reflect differences in leaf structure? Ecoscience 6, 471–474.

[66] Lima CS, Torres-Boeger MR, Carvalho LL, Pelozzo A, Soffiatti P (2013) Sclerophylly in mangrove tree species from South Brazil. Rev Mex Biodiv 84:1159–1166.

[67] Junk WJ, Piedade MTF, Parolin P, Wittmann F, Schöngart J (2010) Ecophysiology, biodiversity and sustainable management of central Amazonian floodplain forests: A synthesis. In: Junk WJ, Piedade MTF, Wittmann F, Schöngart J, Parolin P, editors.Amazonian Floodplain Forests: Ecophysiology, Biodiversity and Sustainable Management.Dordrecht, Heidelberg, London, New York: Springer. pp. 511–540.

[68] Waldhoff D, Parolin P (2010) Morphology and anatomy of leaves. In: Junk WJ, Piedade MTF, Wittmann F, Schöngart J, Parolin P, editors.Amazonian Floodplain Forests: Ecophysiology, Biodiversity and Sustainable Management.Dordrecht, Heidelberg, London, New York: Springer. pp.179–202.

[69] Madeira JA, Maia VC, Monteiro RF (2002) Gall makers (Cecidomyiidae, Diptera) on *Calophyllum brasiliense* Camb. (Clusiaceae): descriptions and biology. Arch Mus Nac (Rio de J) 61:31–48.

[70] Ribeiro KT, Madeira JA, Monteiro RF (1998) Does flooding favour galling insects? Ecol Entomol 23:491–494.

[71] Fagundes MFS, Neves FS, Fernandes GW (2005) Direct and indirect interactions involving ants, insect herbivores and parasitoids on the host plant Baccharis dracunculifolia (Asteraceae). Ecol Entomol 30:28–35.

[72] Mendonça Jr MS (2007) Plant diversity and galling arthropod diversity searching for taxonomic patterns in an animal-plant interaction in the neotropics. Bol Soc Argent Bot 42:347–357.

[73] Fernandes GW, Almada ED, Carneiro MAA (2010) Gall-Inducing Insect Species Richness as Indicators of Forest Age and Health. Environ Entomol 39:1134–1140. 22127163 10.1603/EN09199

[74] Wright MG, Samways MJ (1996) Gall-insect species richness in the African Fynbos and Karoo vegetation: The importance of plant species richness. Biodiv Lett 3:151–155.

[75] Ribeiro SP, Borges PAV, Gaspar C, Melo C, Serrano ARM, et al (2005) Canopy insect herbivores in the Azorean Laurisilva forests: key host plant species in a highly generalist insect community. Ecography 28:315–330.

[76] Carvalho-Fernandes SP (2010) Insetos galhadores associados à família Burseraceae da Reserva Florestal Ducke, Manaus-AM. Unpublished MSc thesis, Instituto Nacional de Pesquisas da Amazônia, Brazil.

