# Supplementary material for: Unexpected High Diversity of Galling Insects in the Amazonian Upper Canopy: The Savanna Out There
Source: PLoS One. 2014 Dec 31;9(12):e114986. doi: 10.1371/journal.pone.0114986 (PMC4281248; doi:10.1371/journal.pone.0114986)
Supplement: S1 Table — Sampling points in the Amazonian forest types, the richness and abundance of galling insects, host plants, and geographical location. (PDF) [file pone.0114986.s001.pdf]

Table S1 Sampling points in the Amazonian forest types, the richness and abundance of galling insects, host plants, and geographical location.

SP	FT	Latitude	Longitude	GIR	GIA	HPS	HPI	TSS	GIR/TSS	HPGen	HPFam
1	*Igapó*	2°43'29.39"S	64°22'44.47"W	25	1805	9	18	10	2.50	9	8
2	*Igapó*	2°43'15.35"S	64°22'43.39"W	16	2822	6	15	8	2.00	5	4
3	*Igapó*	2°41'58.99"S	64°20'58.99"W	35	1717	14	18	14	2.50	11	10
4	*Igapó*	2°28'21.72"S	64°36'43.27"W	17	2190	7	18	9	1.89	7	6
5	*Igapó*	2°29'4.67"S	64°38'25.15"W	36	1025	13	18	14	2.57	10	8
6	*Igapó*	2°28'57.11"S	64°37'48.36"W	34	633	11	17	12	2.83	8	8
7	*Igapó*	2°42'43.99"S	64°21'24.77"W	30	1681	10	16	12	2.50	8	8
8	*Igapó*	2°42'31.61"S	64°20'45.60"W	22	1573	10	12	13	1.69	10	8
9	*Igapó*	2°33'25.81"S	64°41'43.44"W	18	720	10	16	11	1.64	10	7
10	*Igapó*	2°33'39.53"S	64°42'32.87"W	24	1827	11	16	11	2.18	11	9
11	*Igapó*	2°38'50.93"S	64°41'8.34"W	41	2671	15	17	17	2.41	12	8
12	*Igapó*	2°38'35.63"S	64°40'0.66"W	24	3497	11	17	11	2.18	9	8
13	*Igapó*	2°37'55.38"S	64°41'50.75"W	22	1254	9	14	12	1.83	9	8
14	*Igapó*	2°37'32.95"S	64°40'49.15"W	21	579	11	17	11	1.91	11	10
15	*Terra Firme*	2°23'36.0"S	59°50'25.0"W	49	5222	18	20	18	2.72	15	10
16	*Terra Firme*	2°23'22.5"S	59°50'10.7"W	42	6013	16	17	16	2.63	15	11
17	*Terra Firme*	2°26'08.3"S	59°50'14.3"W	42	2154	19	20	20	2.10	17	11
18	*Terra Firme*	2°25'45.8"S	59°49'39.3"W	31	2422	15	17	16	1.94	12	10
19	*Terra Firme*	2°25'58.5"S	59°49'48.6"W	21	516	10	10	10	2.10	10	6
20	*Terra Firme*	2°20'31.2"S	59°57'23.1"W	30	1891	13	13	14	2.14	11	6
21	*Terra Firme*	2°25'52.5"S	59°50'15.7"W	48	3072	16	18	17	2.82	16	9
22	*Terra Firme*	2°20'16.1"S	59°57'27.6"W	44	4860	18	19	18	2.44	14	9
23	*Terra Firme*	2°25'42.0"S	59°50'19.3"W	28	4126	14	17	14	2.00	12	11
24	*Terra Firme*	2°23'58.5"S	59°54'20.0"W	24	2944	12	13	13	1.85	11	7
25	*Terra Firme*	2°23'54.8"S	59°54'30.0"W	17	2719	12	12	12	1.42	11	9
26	*Terra Firme*	2°24'30.2"S	59°52'53.2"W	50	5986	18	20	20	2.50	15	12
27	*Terra Firme*	2°22'29.0"S	59°58'0.1"W	47	4820	21	22	22	2.14	18	13
28	*Terra Firme*	2°22'21.2"S	59°57'53.9"W	60	5310	22	24	22	2.73	16	11
29	*Várzea*	2°59'0.89"S	64°55'35.83"W	53	3502	25	31	33	1.61	23	13
30	*Várzea*	3°3'29.16"S	64°50'56.11"W	36	2005	18	23	28	1.29	16	10
31	*Várzea*	2°49'29.28"S	64°57'12.17"W	22	1032	12	14	17	1.29	12	8
32	*Várzea*	2°47'36.71"S	65°6'15.77"W	30	1430	15	18	19	1.58	14	9
33	*Várzea*	3°1'32.05"S	65°0'22.50"W	35	3392	16	26	17	2.06	15	12
34	*Várzea*	2°57'19.40"S	65°2'56.03"W	19	744	6	11	7	2.71	4	3
35	*Várzea*	2°23'27.35"S	65°20'11.33"W	50	4566	25	29	26	1.92	21	14
36	*Várzea*	2°44'7.44"S	65°13'26.58"W	33	2964	16	22	19	1.74	16	10
37	*Várzea*	2°56'44.99"S	64°32'8.41"W	13	355	7	8	8	1.63	6	5
38	*Várzea*	2°56'21.95"S	64°32'27.78"W	13	823	8	9	9	1.44	7	6
39	*Várzea*	2°45'45.43"S	64°44'16.51"W	26	999	12	13	13	2.00	11	9
40	*Várzea*	2°46'4.15"S	64°43'41.88"W	23	562	11	12	16	1.44	11	10
41	*Várzea*	2°42'36.79"S	64°49'26.94"W	20	3187	12	17	12	1.67	12	10
42	*Várzea*	2°42'39.42"S	64°47'43.33"W	19	683	11	13	14	1.36	10	9

SP: Sampling Points

FT: Forest Type

GIR: Galling Insect Richness

GIA: Galling Insect Abundance

HPS: number of Host Plant Species

HPI: number of Host Plant Individuals

TSS: number of Tree Species Sampled

GIR/TSS: ratio between the galling insect richness and the number of tree species sampled

HPGen: number of Host Plant Genera sampled

HPFam: number of Host Plant Families sampled
