# Supplementary material for: Unexpected High Diversity of Galling Insects in the Amazonian Upper Canopy: The Savanna Out There
Source: PLoS One. 2014 Dec 31;9(12):e114986. doi: 10.1371/journal.pone.0114986 (PMC4281248; doi:10.1371/journal.pone.0114986)
Supplement: S2 Table — Host plant list of the Amazonian gall-forming insects. (PDF) [file pone.0114986.s002.pdf]

**Table S2** Host plant list of the Amazonian gall-forming insects.

*Acacia polyphylla* DC.
*Acacia* sp.
*Acosmium nitens* (Vog.) Yakovl.
*Albizia multiflora* (Kunth) Barn. & Grimes
*Albizia subdimidiata* (Splitg.) Barn. & Grimes
*Allantoma decandra* (Ducke) S.A. Mori, Ya Y.Huang & Prance
*Amanoa gracillima* W.J. Hayden
*Anacardium parvifolium* Ducke
*Anacardium spruceanum* Benth. ex Engl.
*Aspidosperma carapanauba Pichon*
*Aspidosperma excelsum* Benth.
*Aspidosperma* sp. 1
*Blepharocalyx* sp.
*Bocageopsis multiflora* (Mart.) R.E.Fries
*Bocoa viridifolia* (Ducke) R.S. Cowan.
*Brosimum guianensis* (Aubl.) Huber
*Brosimum lactescens* (Moore) C.C.Berg.
*Brosimum parinarioides* Ducke
*Brosimum rubescens* Taub.
*Brosimum utile* subsp. *ovatifolium* (Ducke) C.C. Berg
*Buchenavia grandis* Ducke
*Buchenavia guianensis* (Aubl.) Alwan & Stace
*Buchenavia oxycarpa* (Mart.) Eichler
*Buchenavia parvifolia* Ducke
*Byrsonima* cf. *densa* (Poir.) DC.
*Byrsonima garcibarrigae* Cuatrec
*Byrsonima stipulacea* Juss.
*Calophyllum brasiliensis* Cambess.
*Calyptranthes crebra* McVaugh.
*Calyptranthes cuspidata* Mart ex DC.
*Calyptranthes* sp.1
*Calyptranthes* sp.2
*Calyptranthes* sp.3
*Campsiandra comosa* Benth.
*Campsiandra* sp.
*Caraipa densifolia* Mart.
*Caraipa heterocarpa* Ducke
*Caryocar microcarpum* Ducke
*Caryocar pallidum* A.C.Sm.
*Caryocar villosum* (Aubl.) Pers.
*Caseria sylvestris* Sw. var. *sylvestris*
*Chaetocarpus schomburgkianus* (Kuntze) Pax & Hoffm.
*Cheiloclinium cognatum* (Meiers) A.C. Sm.
*Chimarrhis* cf. *barbata* (Ducke) Bremek.
*Chrysophyllum amazonicum* T.D. Penn.
*Chrysophyllum manaosense* (Aubrév.) T.D. Penn.
*Chrysophyllum sanguinolentum* (Pierre) Baehni
*Chrysophyllum sanguinolentum* subsp. *spurium* (Ducke) T.D. Penn.
*Chrysophyllum ucuquirana-branca* (Aubrév. & Pellegr.) T.D. Penn.
*Chrysophyllum wilsonii* T.D.Penn.
*Clarisia racemosa* Ruiz & Pav.
*Clusia columnaris* Engl.
*Coccoloba* sp.
*Cordia sagotii* I.M.Johnst.
*Corithophora alta* R.Knuth.
*Corythophora rimosa* W.A. Rodrigues
*Couepia caryophylloides* Benoist
*Couepia chrysocalix* (Poepp & Endl.) Benth. ex Hook. f.
*Couepia habrantha* Standl.
*Couepia paraensis* (Mart. & Zucc.) Benth.
*Couepia ulei* Pilg.
*Couma guianensis* Aubl.
*Couratari stellata* A.C.Sm.
*Couroupita guianensis* Aubl.
*Coussapoa trinervia* Spruce ex Mildbr.
*Coussapoa villosa* Poepp. & Endl.
*Cynometra bauhiniaefolia* Benth.
*Cynometra spruceana* Benth.
*Dalbergia inundata* Spruce ex Benth.
*Dialium guianense* (Aubl.) Sandw.
*Dicorynia guianensis* Amshoff
*Dicorynia paraensis* Benth.
*Diospyros capreifolia* Mart. ex Hiern.
*Diospyros cavalcantei* Sothers
*Diospyros* sp.
*Dipteryx magnifica* (Ducke) Ducke
*Duckeodendron cestroides* Kuhlm.
*Duckesia verrucosa* (Ducke) Cuatrec.
*Duguetia argentea* (R.E.Fries) R.E.Fries
*Duguetia quitarensis* Benth.
*Ecclinusa guianensis* Eyma
*Elaeoluma glabrescens* (Mart. & Eichler) Aubrév
*Elaeoluma nuda* (Baehni) Aubrév.
*Endlicheria* sp.
*Endopleura uchi* (Huber) Cuatrec.
*Eperua glabriflora* (Ducke) R.S. Cowan
*Ephedranthus amazonicus* R.E.Fries
*Erisma calcaratum* (Link.) Warm.
*Erisma fuscum* Ducke
*Eschweilera amazoniciformis* S.A.Mori
*Eschweilera apiculata* (Miers) A.C. Smith
*Eschweilera atropetiolata* S.A.Mori
*Eschweilera bracteosa* (Poepp ex O.Berg) Miers
*Eschweilera collina* Eyma
*Eschweilera coriacea* (DC.) S.A. Mori
*Eschweilera micrantha* (O.Berg) Miers
*Eschweilera ovalifolia* (DC.) Nied.
*Eschweilera romeu-cardosoi* S.A.Mori
*Eschweilera* sp.2
*Eschweilera tessmanii* R.Knuth.

**Table S2** Host plant list of the Amazonian gall-forming insects.

*Eschweilera truncata* A.C.Sm.
*Eschweilera wachenheimii* (Benoist) Sandw.
*Eugenia* sp. 6
*Eugenia* sp.1
*Eugenia* sp.2
*Eugenia* sp.4
*Eugenia* sp.5
*Euplassa inaequalis* (Pohl.) Engl.
*Ferdinandusa rudgeoides* (Benth.) Wedd.
*Ficus americana* subsp. *guianensis* (Desv. ex Ham.) C.C.Berg.
*Ficus* cf. *insipida* Willd.
*Ficus* cf. *maxima* Miller
*Ficus* cf. *paraensis* (Miq.) Miq.
*Ficus citrifolia* P.Miller
*Ficus duckeana* C.C.Berg & Ribeiro
*Ficus trigona* L.f.
*Geissospermum argenteum* Woodson
*Goupia glabra* Aubl.
*Guarea* sp.1
*Guarea* sp.2
*Guatteria* cf. foliosa Benth.
*Guatteria* cf. riparia R.E.Fries
*Guatteria citriodora* Ducke
*Guatteria discolor* R.E.Fries
*Guazuma ulmifolia* Lam.
*Gustavia hexap*etala (Aubl.) Sm.
*Heisteria laxi*flora Engl.
*Heisteria spruceana* Engl.
*Hevea guianensis* Aubl.
*Hevea spru*ceana (Benth.) Müll. Arg.
*Himatanthus sucuuba* (Spruce ex Müll.Arg.) Woodson
*Hirtella racemosa* Lam.
*Homalium racemosum* Jacq.
*Humiriastrum cuspidatum* var. *glabriflorum* (Ducke) Cuatrec.
*Hydrochorea marginata* (Benth.) Barn. & Grimes
*Hydrochorea marginata* (Benth.) Barn. & Grimes var. *marginata*
*Hyeronima* sp.
*Hymenolobium nitidum* Benth.
*Hymenolobium sericeum* Ducke
*Ilex inundata* Poepp. ex Reissek
*Ilex* sp.
*Inga capitata* Desv.
*Inga* cf. *alba* (Swartz.) Willd
*Inga* cf. *nobilis* Willd.
*Inga cordatoalata* Ducke
*Inga disticha* Benth.
*Inga edulis* Mart.
*Inga ingoides* (Rich.) Willd.
*Inga macrophylla* Humb.&Bonpl. ex Willd.
*Inga marginata* Willd.
*Inga paraensis* Ducke
*Inga rubiginosa* (Rich.) DC.
*Inga* sp.1
*Inga* sp.2
*Inga thibaudiana* DC.
*Inga umbellifera* (Vahl) DC.
*Lacistema aggregatum* (Berg.) Rusby
*Laetia corymbulosa* Spruce ex Benth.
*Lecointea amazonica* Ducke
*Lecythis* aff. *chartacea* Berg.
*Lecythis holcogyne* (Sandw.) S.A.Mori
*Lecythis prancei* S.A. Mori
*Lecythis zabucajo* Aubl.
*Licania* aff. *majuscula* Sagot
*Licania apetala* (E.Mey) F. var. *apetala* (Benth.) Prance
*Licania apetala* (E.Mey.) Fritsch
*Licania* cf. *caudata* Prance
*Licania* cf. *kunthiana* Hook. f.
*Licania* cf. *pallida* Spruce ex Sagot
*Licania* cf. *parviflora* Benth.
*Licania egleri* Prance
*Licania fanshawei* Prance
*Licania heteromorpha* Benth.
*Licania heteromorpha* var. *glabra* (Mart. ex Hook.f.) Prance
*Licania impressa* Prance
*Licania licaniiflora* (Sagot) Blake
*Licania micrantha* Miq.
*Licania mollis* Benth.
*Licania octandra* subsp. *pallida* (Hook.f.) Prance
*Licania pallida* Spruce ex Sagot
*Licania prismatocarpa* Spruce ex Hook.f.
*Licania rodriguesii* Prance
*Licania sandwithii* Prance
*Licania unguiculata* Prance
*Licaria chrysophylla* (Meisn.)Kosterm.
*Licaria pachycarpa* (Meisn.) Kosterm.
*Licaria* sp.
*Luehea cymulosa* Spruce ex Benth.
*Mabea nitida* Spruce ex Benth.
*Mabea piriri* Aubl.
*Mabea* sp.2
*Mabea speciosa* Müll. Arg.
*Mabea subsessilis* Pax & K. Hoffm.
*Maclura tinctoria* (L.) D.Don ex Steud. subsp. *tinctoria*
*Macrolobium acaciifolium* (Benth.) Benth.
*Macrolobium angustifolium* (Benth.) R.S.Cowan
*Macrolobium multijugum* (DC.) Benth.
*Malouetia* aff. *flavescens* (Willd. ex Roem. & Schult.) Müll.Arg.
*Malouetia duckei* Markgr.
*Malouetia tamaquarina* (Aubl.) A.DC.

**Table S2** Host plant list of the Amazonian gall-forming insects.

*Manilkara bidentata* (A. DC.) A. Chev.
*Manilkara cavalcantei* Pires & W.A. Rodrigues ex T.D. Penn.
*Manilkara huberi* (Ducke) A. Chev.
*Manilkara inundata* (Ducke) Ducke
*Maquira coriacea* (H.Karst) C.C. Berg
*Maquira sclerophylla* (Ducke) C.C.Berg.
*Matayba arborescens (*Aubl.) Radlk.
*Maytenus ebenifolia* Reissek
*Mesilaurus itauba* (Meisn.) Taubert ex Mez
*Miconia burchellii* Triana
*Miconia elaeagnoides* Cogn.
*Miconia spichigeri* Wurdack
*Micrandra siphonioides* Benth.
*Micrandropsis scleroxylon* (W.A.Rodrigues) W.A.Rodrigues
*Micropholis casiquiarensis/mensalis* Aubrév.
*Micropholis egensis* (A.DC.) Pierre
*Micropholis guyanensis* (A. DC.) Pierre
*Micropholis guyanensis* (A.DC.) Pierre subsp. *guyanensis*
*Micropholis guyanensis* subsp. *duckeana* (Baehni) T.D. Penn.
*Micropholis venulosa* (Mart. & Eichler) Pierre
*Minquartia guianensis* Aubl.
*Moronobea* sp. 1 *
*Mouriri angulicosta/duckeana /duckeanoides* Morley *
*Mouriri dimorphandra* Morley
*Mouriri ficoides* Morley
*Mouriri grandiflora* DC.
*Mouriri guianensis* Aubl.
*Mouriri nigra* (DC.) Morley
*Myrcia amazonica* DC.
*Myrcia* sp.
*Nealchornea yapurensis* Huber
*Nectandra amazonum* Nees
*Nectandra* sp.
*Ocotea aciphylla* (Nees & Mart.) Mez
*Ocotea argyrophylla* Ducke
*Ocotea cernua* (Nees) Mez
*Ocotea cinerea* van der Werff
*Ocotea cujumary* Mart.
*Ocotea cymbarum* Kunth
*Ocotea glomerata* (Nees) Mez
*Ocotea longifolia* Kunth
*Ocotea mattogrossensis* Vattimo-Gil
*Ocotea nigrescens* Vicentini
*Ocotea percurrens* Vicentini
*Osteophloeum platyspermum* (Spruce ex A.DC.) Warb.
*Ouratea ferruginea* Engl.
*Ouratea* sp.
*Oxandra polyantha* R.E.Fries
*Oxandra riedeliana* R.E.Fries
*Paramachaerium ormosioides* (Ducke) Ducke
*Parinari parvifolia* Sandw.
*Parkia nitida* Miq.
*Peltogyne excelsa* Ducke
*Peltogyne paniculata* Benth.
*Pera bicolor* (Klotzsch) Müll. Arg.
*Picramnia juniniana* J.F.Macbr.
*Piranhea trifoliata* Baill.
*Pourouma tomentosa* subsp. *apiculata* (Spruce ex Benoist) C.C.Berg & Heusden
*Pouteria reticulata* (Engl.) Eyma.
*Pouteria* aff. *cuspidata* (A.DC.) Baehni
*Pouteria anomala* (Pires) T.D.Penn.
*Pouteria biloculares* (Winkl.) Baehni
*Pouteria caimito* (Ruiz & Pav) Radlk.
*Pouteria cladantha* Sandw.
*Pouteria cuspidata* (A.DC.) Baehni
*Pouteria durlandii* (Standl.) Baehni
*Pouteria engleri* Eyma
*Pouteria eugeniifolia* (Pierre) Baehni
*Pouteria fimbriata* Baehni
*Pouteria freitasii* T.D.Penn.
*Pouteria fulva* T.D.Penn.
*Pouteria glomerata* (Miq.) Radlk.
*Pouteria gomphiifolia* (Mart. ex Miq.) Radlk.
*Pouteria guianensis* Aubl.
*Pouteria jariensis* Pires & TD Penn.
*Pouteria minima* T.D. Penn.
*Pouteria* sp.1
*Pouteria* sp.2
*Pouteria speciosa* (Ducke) Baehni
*Pouteria stylifera* T.D. Penn.
*Protium altsonii* Sandw.
*Protium hebetatum* Daly
*Protium polybotryum* (Turcz.) Engl.
*Pseudopiptadenia suaveolens* (Miq.) Grimes
*Psidium acutangulum* DC.
*Psychotria gracilenta* Mull.Arg.
*Pterocarpus amazonicus* Huber
*Pterocarpus officinalis* Jacq.
*Pterocarpus* sp.1
*Pterocarpus* sp.2
*Qualea clavata* Stafl.
*Qualea paraensis* Ducke
*Quiina rhytidopus* Tul.
*Sacoglottis mattograssensis* Malme var. *subintegra* (Ducke) Cuatrec.
*Sacoglottis* sp.1
*Sacoglottis* sp.2
*Sapium glandulosum* (L.) Morong
*Sclerolobium* cf. *chrysophyllum* Poepp & Endl.
*Sclerolobium melanocarpum* Ducke
*Sclerolobium micropetalum* Ducke

**Table S2** Host plant list of the Amazonian gall-forming insects.

*Sclerolobium* sp. 2 *
*Sclerolobium* sp. 4 *
*Scleronema micranthum* Ducke
*Sextonia rubra* (Mez) van der Werff.
*Sloanea eichleri* K.Schum.
*Sloanea fendleriana* Benth.
*Sloanea floribunda* Spruce ex Benth.
*Sloanea guianensis* (Aubl.) Benth.
*Sloanea laurifolia* (Benth.) Benth.
*Sloanea* sp.1
*Sorocea duckei* W.C. Burg.
*Sorocea* sp.
*Spondias lutea* L.
*Sterculia speciosa* K.Schum.
*Stryphnodendron paniculatum* Poepp. & Endl.
*Stryphnodendron racemiferum* (Ducke) Rodr.
*Styrax* cf. *guianensis* A. DC.
*Swartzia arborescens* (Aubl.) Pittier
*Swartzia oblanceolata* Sandw.
*Swartzia polyphylla* DC.
*Swartzia recurva* Poepp.
*Swartzia reticulata* Ducke
*Swartzia schomburgkii* Benth.
*Swartzia ulei* Harms
*Symeria paniculata* Benth.
*Tabebuia barbata* (E.Mey) Sandw.
*Tachigali* cf. *myrmecophila* (Ducke) Ducke
*Tachigali pumblea* Ducke
*Tachigali* sp.
*Tachigali venusta* Dwyer
*Tapirira* sp.2
*Tapura amazonica* Poepp. & Endl.
*Tapura guianensis* Aubl.
*Taralea oppositifolia* Aubl.
*Terminalia argentea* Mart.
*Trichilia* sp.
*Triplaris longifolia* Huber
*Trymatococcus amazonicus* Poepp & Endl.
*Unonopsis guatterioides* (A.DC.) R.E. Fries
*Vantanea micrantha* Ducke
*Vantanea parviflora* Lam.
*Vatairea guianensis* Aubl.
*Virola guggenheimii* W.A. Rodrigues
*Virola michelii* Heckel
*Virola surinamensis* (Rol. ex Rottb.) Warb.
*Xylopia aff. emarginata* Mart.
*Xylopia calophylla* R.E.Fries
*Xylopia cf. benthamii* R.E.Fries
*Xylopia polyantha* R.E.Fries
*Zanthoxylum rhoifolium* Lam.
*Zygia cataractae* (Kunth) L.Rico
*Zygia racemosa* (Ducke) Barn. & Grimes
*Zygia* sp.1
*Zygia* sp.2
*Zygia* sp.3
